# The Asymmetric Impact of War: Resilience, Vulnerability and Implications for EU Policy

**DOI:** 10.1007/s10272-022-1049-2

**Published:** 2022-06-07

**Authors:** Giuseppe Celi, Dario Guarascio, Jelena Reljic, Annamaria Simonazzi, Francesco Zezza

**Affiliations:** 1 Department of Economic, Università degli Studi di Foggia, Largo Papa Giovanni Paolo II, 1, 71121 Foggia, Italy; 2 Department of Economic and Law, Sapienza University of Rome, Via Del Castro Laurenziano 9, 00161 Rome, Italy

**Keywords:** F51, O52

## Abstract

In 2019, over 96% of EU27 oil needs, nearly 90% of natural gas and over 43% of solid fuels were met by net imports, with the largest share coming from Russia (35% of oil, 40% of natural gas and 20% of solid fuels consumed in EU27). The decline in the share of oil since 2016 was more than off-set by the increase in gas and solid fuels.

Contrary to what Jean Monnet imagined, the European Union does not seem to become stronger and more integrated "crisis after crisis". Recent history attests to how symmetric shocks systematically translate into divergence and polarisation, both between and within member states (Celi et al., 2018; Gräbner et al., 2020). This was the case of the financial crisis of 2008, which highlighted the contradictions of the fiscal and monetary policy setting of the eurozone while the structural gap between the centre and the periphery continued to widen (Celi et al., 2019). The same goes for the COVID-19 pandemic (Ceron and Palermo, 2022). Where deflationary policies and spending cuts have been more prevalent, such as in the southern periphery (Storm, 2019), death rates and socio-economic costs have skyrocketed (Prante et al., 2020). Likewise, the renunciation of industrial policy in the name of export-driven competitiveness has contributed to undermining the EU's production capacity for a number of essential products, starting with vaccines (Celi et al., 2020). This is evident in a growing dependence on the United States, China and, more generally, on multinationals leading key technology domains (e.g. big pharma, big tech).

The Russia-Ukraine war is no exception. The channels through which it will influence the economy are many (Pisani-Ferry, 2022; Astrov et al., 2022). First, there is an exponential growth of uncertainty, which negatively affects consumption and investment, with depressive effects on GDP and employment: the longer the war lasts, the greater and more persistent its effects will be. Second, sanctions against Russia are fueling tensions over energy and commodity prices. Rising energy costs will cause companies to reduce production, postpone investment and reduce employment. Likewise, inflation can diminish the purchasing power of households, particularly those at the bottom of income distribution, further depressing aggregate demand and GDP. Adding to the bottlenecks caused by COVID-19 (Baldwin and Freeman, 2021), the war is further destabilising global value chains (GVCs), with the shortage of key intermediate inputs that increase production costs and put entire industries at risk. In the medium term, this could accelerate a process of deglobalisation (Dadush, 2022) – reshoring or "friendshoring" – which could lead to a "decoupling of the global trading system into two blocs – a US-centric and a China-centric bloc" (Bekkers and Góes, 2022). If this is the plausible scenario, sustaining incomes, strengthening internal markets, and recovering technological and productive sovereignty are considered the foremost political priorities.

## What are the expected consequences for the European economy?

European countries are affected differently by the war but in ways that transcend the traditional core-periphery division. So far, the German manufacturing core (GMC, made up of Germany and the Visegrád countries) stands out as the most resilient part of the EU economy in the face of a crisis (Celi et al., 2018). This time, the degree of economic vulnerability associated with the weight of energy-intensive manufacturing in the economy, dependence on energy production and diversification of supply determine the costs in terms of inflation and growth. In this respect, the GMC and Italy – the southern periphery's major economy – share a significant degree of vulnerability: A large share of energy-intensive manufacturing and a strong import dependency on Russian energy reduce the room for adjustment and make the risk of a prolonged recession greater. Diversification of energy sources and composition, in particular renewables, will only work in the medium term. Equally serious is dependence on Russian and Ukrainian key raw materials and intermediate goods (e.g.

* This essay is part of the activities funded by the Dezernat Zukunft Foundation in the context of the European Macro Policy Network project. All the usual disclaimers apply.

**Giuseppe Celi**, University of Foggia, Italy.

**Dario Guarascio**, Sapienza University of Rome, Italy.

**Jelena Reljic**, Sapienza University of Rome, Italy.

**Annamaria Simonazzi**, Sapienza University of Rome, Italy.

**Francesco Zezza**, Sapienza University of Rome, Italy.

iron, cereals, fertilisers): The risks of bottlenecks and supply restrictions feed sector-specific inflationary shocks, which are easily transmitted to the whole economy. Key German industries, such as the automotive industry, are particularly vulnerable to the disruption of specific supply chains.[1]

But why did the GMC and Italy develop such a strong dependence on fossil fuels and, in particular, on Russian gas? A possible explanation can be found in the model of growth and the long-term evolution of these economies. First, Germany's mercantilist strategy, which aimed at promoting exports to the detriment of domestic demand (both consumption and investment), required minimising costs in order to maximise export competitiveness. Cheap Russian gas was an important element of this strategy, as was the relocation of German industry to the East. For the GMC, strengthening its links with the Russian economy, both in terms of energy supply and in terms of creating a market for its exports, represented an obvious strategic development. Economic interests, as well as the illusion behind the *doux commerce* doctrine, may explain the lack of diversification efforts as ties to the Russian energy sector grew stronger. Similar arguments hold true for Italy: its historical ties with the Russian energy sector (e.g. relations between the Italian state-owned oil company Eni and its Russian counterpart Gazprom have strengthened since the 1990s)[2] and the importance of Russia as an exporting market for Italian products, both capital and final goods.

Are we therefore overcoming the core-periphery division? Not exactly. The relative initial vulnerability of member states matters as much as their position within European (and global) production and trade networks. As Landesmann (2020) noted, periphery countries are more vulnerable in many ways, and the war may increase the structural divide. Their higher share of low-wage precarious workers (for a detailed analysis, see Eurofound, 2021), their weaker welfare systems and their smaller fiscal space[3] are bound to increase the social costs of the crisis. Furthermore, the periphery has a higher share of micro and small enterprises (in Italy, firms with less than ten employees account for about 95% of the total). These enterprises, characterised by low capitalisation and limited organisational capabilities, have poor resilience to crises. Their fragility calls for more extensive government support. A perspective that becomes even more gloomy in the case of a return to fiscal austerity, monetary restraint and a "wartime industrial policy", that is, more weapons and less welfare.

In what follows, we assess the economic implications of the Russia-Ukraine war focusing on member states' relative vulnerability. Relying on a heterogeneous set of data sources, key channels are considered: employment share of energy-intensive industries; import dependency with respect to energy goods (oil, coal and gas), key raw materials and intermediate goods; and production link-

Figure 1

**Employment in high and medium-high energy-intensive sectors in Europe, 2019**

% of total employment

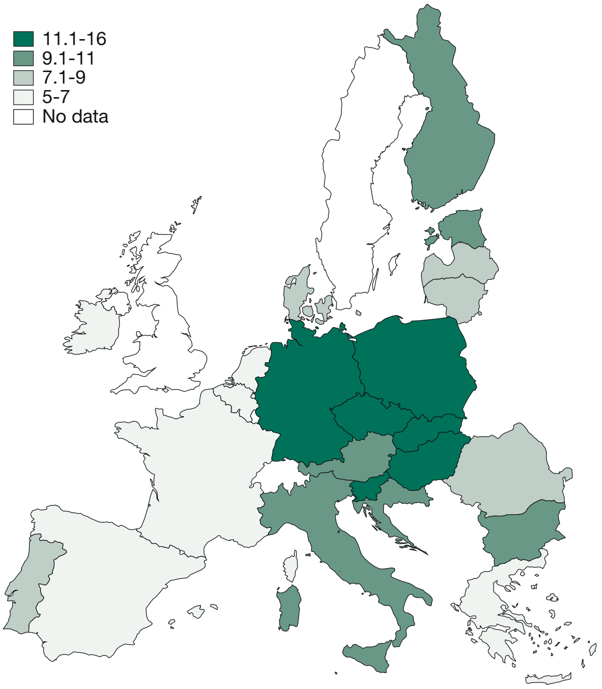

Notes: To define energy-intensive industries, we rely on the Eurostat energy balances ranking sectors according to the ratio between the amount of energy used in that sector and total final energy consumption. We classified industries with an above-the-median ratio as energy-intensive. Once we identified the set of energy-intensive industries (chemical and petrochemical; iron, steel and non-ferrous metals; non-metallic minerals; paper, pulp and printing; food, beverages and tobacco; machinery), we computed their relative employment share for each EU member state. The taxonomy fits all EU member states with the exception of the Baltic countries, where the "wood and wood products" sector has the highest energy intensity.

Source: Authors' elaboration on Eurostat data.

1 Interruption of cable production in Ukraine has prompted Volkswagen and other car producers to stop production at some plants (Campbell and Miller, 2022).

2 The collaborations between the Italian energy giant and its Russian counterpart have never stopped. Among the most important is the 1,200-kilometre Blue Stream pipeline, which connects Russia and Turkey, crossing the Black Sea. Since 1999, Eni and Gazprom have shared ownership of the offshore section of the pipe, through the Blue Stream Pipeline Company B.V., registered in the Netherlands.

3 This is particularly true for Greece and Italy, where the public debt grew exponentially as a consequence of austerity first, and then the COVID-19 crisis. According to Eurostat data, in 2020 the debt-to-GDP ratio in the southern periphery – Greece, Italy, Spain and Portugal – reached an average of 150% against 90% in the EU27.

ages. After mapping the (asymmetric) vulnerability of the European economies, we conclude by discussing the potential risks of adopting a wartime agenda for the EU economic policy.

## Assessing the asymmetric impact of the Russia-Ukraine war

Two distinct but related aspects matter for the assessment of vulnerability to war: the EU's dependence on imports from Russia and Ukraine and the differential impact on individual countries. The latter refers to the relative importance of the most energy-intensive sectors in terms of employment and value added.

### Employment in energy-intensive industries

To assess the relative vulnerability of EU economies to energy shocks, we computed the relative weight of energy-intensive industries for each EU country and the share of workers employed in them (Figure 1). Member states that, other things being equal, show a relatively high share of employment in energy-intensive industries tend to be more exposed to the risk of unemployment and recession. While the highest shares are found in Czechia and Slovenia, at 15.7% and 13.7% respectively, employment in energy-intensive industries also represents over 11.5% of total employment in Germany (equal to 5.3 million workers) and 9.5% in Italy (2.3 million workers). A similar picture emerges if we look at the share of value added generated by such an industry cluster. As anticipated, a strong (and new, compared to previous crises) industry-related divide seems to emerge in Europe, partly reflecting the different importance of the manufacturing industry in the economy: Germany, Austria, Italy and a large part of the eastern periphery on the one side; and Belgium, France, Spain, and the Netherlands on the other.

### Europe's dependence on Russian fossil fuels

The share of energy-intensive industries is only one of the factors that can potentially affect the relative vulnerability of member states. Equally important is the dependence on the import of energy goods. Under current conditions, this is especially true if imports come from Russia. Over the past decade (except in 2020, when COVID-19 hit demand and imports), the dependence of the EU27 has grown across the spectrum of fossil fuels: natural gas, oil and petroleum products and solid fuels (see Figure 2, left-hand panel). The same goes for dependence on Russia (Figure 2, right-hand panel). In 2019, over 96% of EU27 oil needs, nearly 90% of natural gas and over 43% of solid fuels were met by net imports, with the largest share coming from Russia (35% of oil, 40% of natural gas and 20% of solid fuels consumed in EU27).[4] The decline in the share of oil since 2016 was more than offset by the increase in gas and solid fuels.

However, the aggregate figures conceal important heterogeneities between countries. Figure 3 delves into this heterogeneity based on Eurostat's import dependency

4 The highest share of natural gas imports (in total EU imports) comes from Russia (38%), followed by Norway (15%) and Algeria (7%). Russia is also the largest exporter of solid fossil fuels to the EU27, with 41% of total imports, followed by the US with 16% and Australia with 12%. Russia is equally important in oil, with a share of 23% of the EU's imports, followed by Saudi Arabia (6%) and the US (5%).

Figure 2
**EU27 dependency on energy imports**

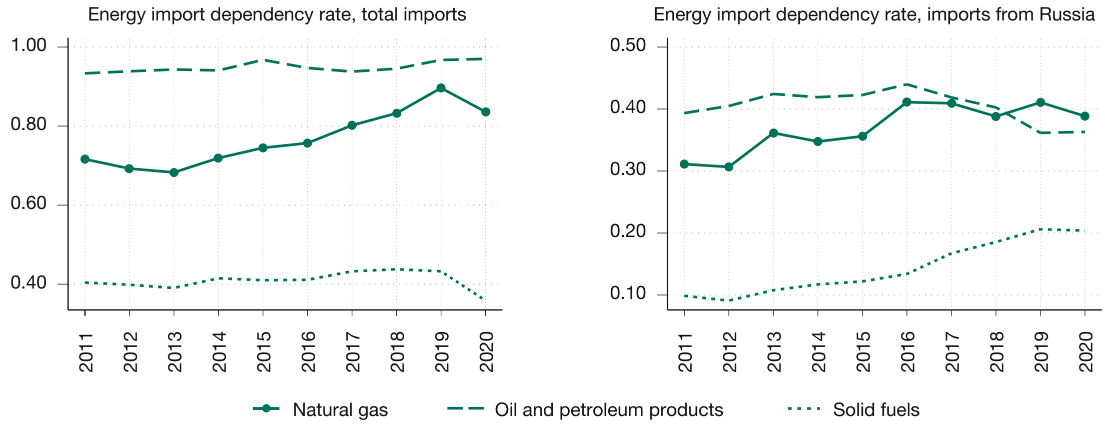

Note: The left-hand panel shows the share of the EU27's energy net imports in gross available energy, while the right-hand panel exhibits the share of EU27 net imports from Russia in gross available energy.

Source: Authors' elaboration on Eurostat Energy data.

Figure 3
**EU member states' dependency on energy imports from Russia, 2019**
Index

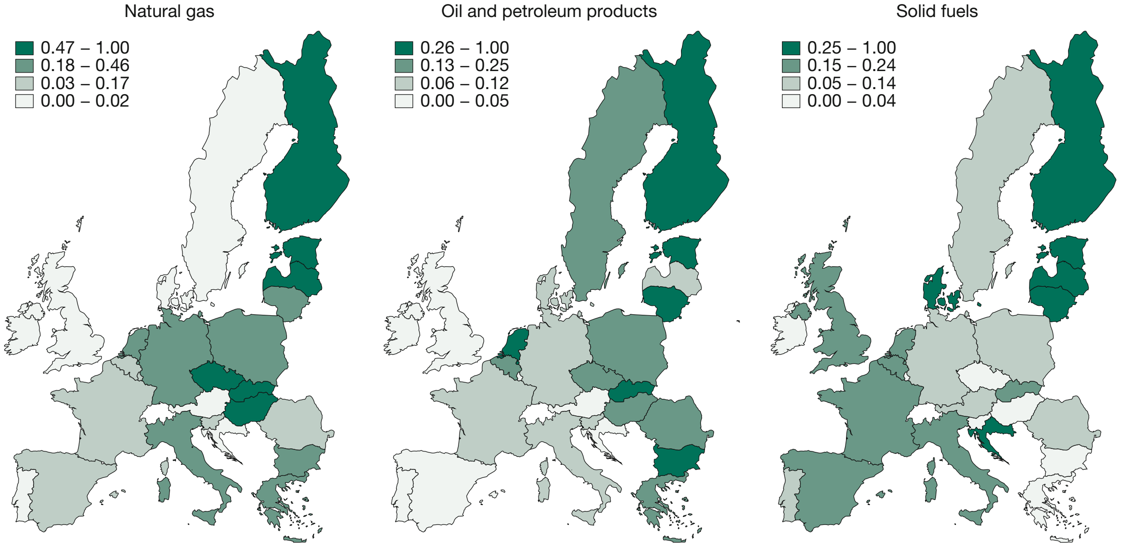

Notes: The energy import dependency rate for each country is defined as the share of net energy imports (imports minus exports) in total available energy – separately for natural gas, oil and petroleum products, and solid fossil fuels. We standardise indicators on a scale of 0 to 1, where zero stands for the lowest position in the ranking and 1 for the highest. The distribution of the indicator is then divided into quartiles to compare the relative position of member states.

Source: Authors' elaboration on Eurostat data.

indicator. A clear East-West divide appears to be emerging. Particularly strong when it comes to gas and oil: Hungary, Czechia and Slovakia obtain virtually all of their natural gas imports from Russia, followed by Finland, Estonia and Latvia, accounting for more than 80% of their natural gas needs. Germany and Italy rely heavily on natural gas and, though belonging to the second quartile of the dependency indicator, they are the biggest importers of Russian gas in volume terms. As for solid fuels, Poland and Germany emerge as more autonomous, partly due to their domestic production.

This preliminary evidence gives an idea of the significant, albeit asymmetrical, costs that EU economies will have to bear if the crisis lasts or worsens (e.g. a full Russian gas embargo). These costs would be much higher, as many scholars have pointed out (see, among others, Pisani-Ferry, 2022), for countries that are more dependent on Russian natural gas, such as Germany and Italy.[5] It also shows how the "new divide" emerging in Europe appears to be strongly associated with a country's industrial specialisation (i.e. relative size of manufacturing, heavy and energy-intensive industries) as well as the extent of its ties to Russia. The only way to reduce vulnerability is to diminish dependence on fossil fuels and diversify sources. Here too, however, a relevant asymmetry can be observed. Although dependence can be reduced by increasing the use of renewable energy, we observe that the most dependent and therefore most vulnerable countries also tend to rank low in terms of the share of energy consumption from renewable sources in gross final energy (see Figure 4).

Industrial specialisation, geographical proximity and historical ties with a gas-rich country such as Russia may explain the poor performance of Eastern European countries in terms of renewables. For countries such as Germany and Italy, in turn, the need to promote an export-oriented, energy-intensive manufacturing sector could have played a significant role. In the short run, the energy crisis could put the brakes on environment-friendly policies; however, a positive and unintended consequence of these tragic events could be the acceleration of the green transition, with an EU-wide industrial policy more focused on addressing current energy vulnerabilities.

5 In fact, although diversification is somewhat more feasible in the case of oil and solid fuels, it will undoubtedly be far more challenging in the case of gas.

Figure 4

**Natural gas import dependency and share of renewables in EU member states, 2019**

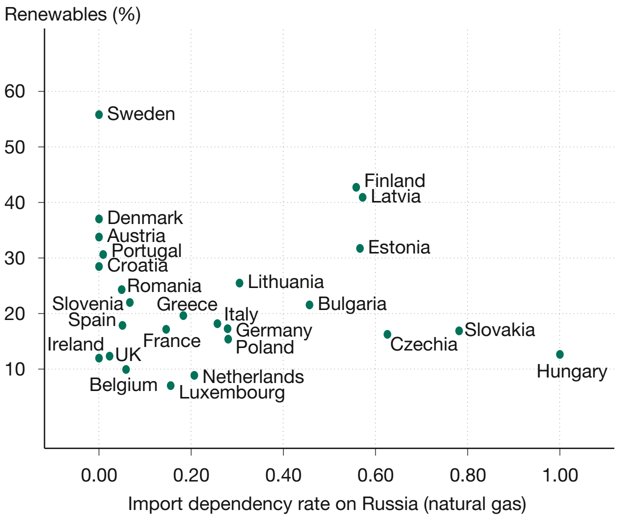

Notes: The indicator of the import dependency rate on Russian natural gas has been standardised to a common scale within the range from 0 to 1. Instead, the renewables share stands for the share of energy consumption from renewable sources in gross final energy.

Source: Authors' elaboration on Eurostat data.

## Exposure to disruptions in GVCs

The war is going to further disrupt value chains. To assess the vulnerability of Europe (and individual member states), we look at the interdependence between Russia and the EU as measured by OECD's GVC backward and forward participation indicators (Figure 5a).[6] While forward participation remains fairly stable over the observed period (averaging around 0.5%), the level of backward participation has nearly tripled: from 0.5% in 1995 to 1.4% in 2018 (though declining after 2011). In other words, 1.4% of the EU27 gross exports in 2018 relied on value added produced in Russia.

Once again, European countries differ in their degree of interaction with the Russian economy (Figure 5b). Bulgaria, Greece, Finland, Slovakia, Latvia and Lithuania stand out with the highest level of backward participation, with values exceeding 4% of exports. Over the period 1995-2018, most countries are above or near the pink line (zero rate of change), suggesting that backward linkages with Russia increased or remained stable, with only the Baltic countries and Slovakia recording a decrease, albeit from a high value. Thus, the costs of a permanent disruption of Russian-related supply chains would spread rather unevenly.

6 The backward participation indicator is defined as the percentage share of value added produced in country j (Russia, in our case) embodied in gross exports of country i (the EU and its member states, in our case) over total gross exports of country i. Basically, the backward participation indicator measures the relative importance of country j's productions for country i's exports. In turn, the forward participation indicator is the share of country i's (the EU and its member states) domestic value added embodied in j's (Russia) gross exports over its total gross exports. This measures the importance of i's productions for j's final exports.

Figure 5a

**Interdependence between Russia and the EU, 1995-2018**

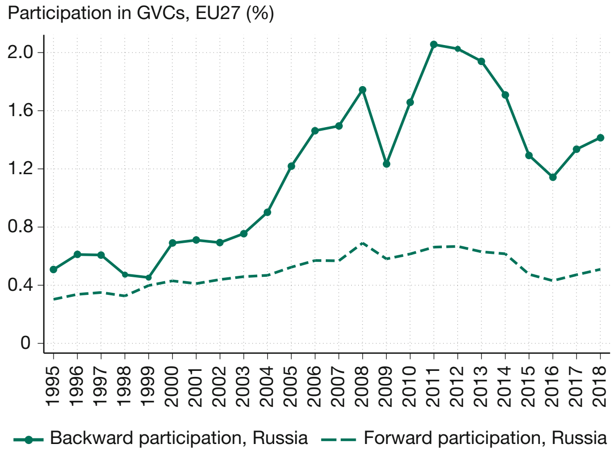

Source: Authors' elaboration on OECD-TIVA.

Figure 5b

**Interdependence between Russia and the EU member states**

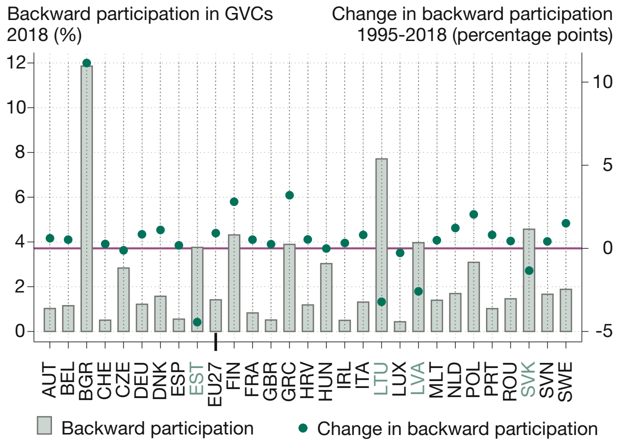

Note: The left-hand side axis refers to the level of backward participation in 2018, while the right-hand side axis refers to long-term changes over the period 1995-2018. The highlighted countries recorded a decrease.

Source: Authors' elaboration on OECD-TIVA.

Figure 6
**The share of imports from Russia in total imports, 2019 (top three products)**
in %

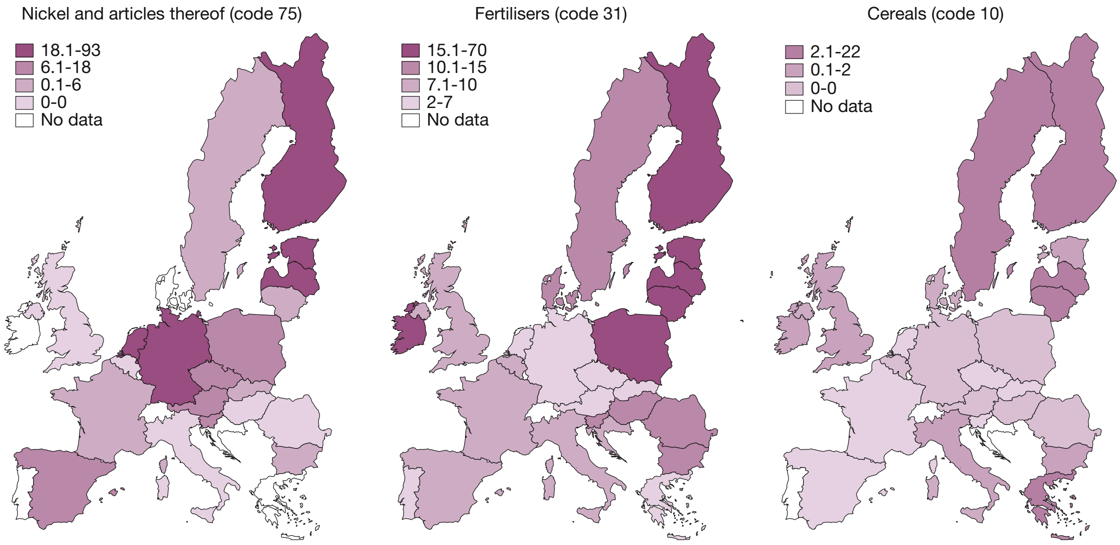

Note: In the case of cereals, half of the countries do not import from Russia.

Source: Authors' elaboration on Comtrade data.

Aggregate figures can hide sector-specific bottlenecks. Even a low aggregate degree of interdependence will not shield from major disruption if there is a strong dependence on imports of specific (strategic) goods. For example, despite Germany's relatively low backward participation indicator with Russia (Figure 5b), the German automotive sector is particularly vulnerable to the disruption of specific goods, such as palladium (used in catalytic converters) and nickel ore (used in lithium-ion car batteries).[7]

Although in 2019 Russian exports accounted for only 2.3% of total world exports, figures were much higher for some specific non-energy, key commodities: fertilisers accounted for 14.3% of global exports, nickel and articles thereof 11.3%, and cereals 7.2%. These three commodities, which play an important role for agricultural and industrial production, weigh differently in EU countries' imports (Figure 6), and their shortage could cause domino effects. In the case of nickel, for example, an input crucial for the production of batteries for electric vehicles, the highest share is recorded in Germany and its manufacturing core, as well as in Spain and the Baltics – countries where the car industry plays a significant role. Enduring shortages may cause interruptions in car production in the short run, and slow down the green transition in the medium term. Fertilisers and cereals are crucial for agriculture, with fallout effects in other sectors, such as cattle breeding and the food industry.

Cross-country interdependence should also not be underestimated, as disruptions taking place in a specific country may be rapidly transmitted to other economies, especially if some countries operate as brokers (i.e. play the role of intermediaries in the trade of specific goods) or process and export strategic intermediate goods. Similar to the increase in energy prices, higher prices of cereals and fertilisers passed on to food can affect people at the bottom of the income distribution (Mitchell et al., 2022), in addition to being deleterious for lower-income countries, which depend on these products for survival. These negative outcomes may be reinforced if the impact of war and sanctions cause a long stagflation.

## What impact on the EU's economic policy?

Russia's invasion of Ukraine, and the consequent sanctions, have unleashed supply and demand shocks, slashing growth, fueling inflation and raising new challenges for the EU's fiscal and monetary policy. The member countries more dependent on fossil fuels are going to suffer more – which explains their opposition to including oil and gas in sanctions. These economies are the EUs' "manufacturing heart", therefore their hardships will be inevitably passed on, in varying degrees, to the whole Union. The first to be hit is likely to be the eastern periphery – which is closely

7 Similar problems concern imports of cables produced in Ukraine (Simchi-Levi and Haren, 2022).

integrated with German industry (Stehrer and Stöllinger, 2015) but has also established ties with Russia – and a portion of the southern periphery that is part of German value chains (Simonazzi et al., 2013).

With COVID-19 still progressing, the need to assist households and businesses in this new crisis will weigh on public budgets and feed on debt. If preventing business bankruptcy is necessary in order to restart the economy, once the recovery sets in, the highly heterogeneous fiscal stance of the member states is likely to affect their capacity to support their economies. The debtor countries could face a more severe challenge, depending on the macroeconomic policy that the EU governing bodies will decide to implement. Although fiscal rules have been frozen for the current year, the growth of public debt – made worse by the commitment to increase military spending – may alarm creditor countries and convince them to resume austerity measures for the whole Union. The definition of the fiscal rules for the future is as important as the policy mix (fiscal, monetary and industrial policies) implemented in the current environment for the survival of the Union.

The war has undoubtedly made the definition of policies more difficult. Rising inflation presents the ECB with a conundrum. While there is a consensus that interest-rate hikes are not appropriate to address price rises due to supply shocks, the need for ECB's credibility to moderate inflationary expectations earns consideration. A central bank that is perceived as being committed to protecting its mandate, it is argued, can contain inflation at a lower economic cost, since the expectation that adequate policy action will be taken is itself stabilising. Such credibility is vital for the conduct of monetary policy. If continuing the path of policy normalisation is therefore the appropriate course of action, its speed will depend on the economic fallout from the war, and the severity and persistence of the inflation shock.

This leaves the issue of the debt management unresolved. It is true that real interest rates are still negative. However, without a firm commitment by the ECB, interest rates on government bonds in debtor countries (namely, the southern periphery) could rise abruptly and steeply, and a contagious panic could set in, forcing the ECB to rashly reverse its policy on bond purchases (Heimberger, 2022). The monetary stance will affect fiscal policy: Euro area governments face a trade-off between business cycle stabilisation and debt sustainability.

The war marks a turning point for the global geopolitical order. Globalisation, already affected by the US-China trade conflict and the disruption following the pandemic, could be in retreat. Deglobalisation may not mean a new age of autarky like in the 1920s and 1930s. Rather, as mentioned above, we could see the formation of international economic blocs (Dadush, 2022). European countries, traditionally dependent on (global) exports, might have to readjust their growth model by giving greater weight to the domestic market. In this context, the need for a "Fortress Europe" can in fact change their macroeconomic stance. A result that is not necessarily a happy ending, and that could have certainly been achieved in less dramatic circumstances.
